# Effects of Induction Plasma Spheroidization on Properties of Yttria-Stabilized Zirconia Powders for Thermal Barrier Coating Applications

**DOI:** 10.3390/ma17071518

**Published:** 2024-03-27

**Authors:** Haoran Peng, Yueguang Yu, Tianjie Shi, Botian Bai, Zheng Yan, Kang Yuan

**Affiliations:** 1 School of Materials Science and Engineering, University of Science and Technology Beijing, Beijing 100083, China; weixuan2007@163.com; 2 BGRIMM Technology Group, Beijing 100160, China; shi981204@163.com (T.S.); ibaibotian@163.com (B.B.); z_yan0926@163.com (Z.Y.); yuankang@bgrimm.com (K.Y.); 3 BGRIMM Advanced Materials Science & Technology Co., Ltd., Beijing 102206, China; 4 China Iron & Steel Research Institute Group, Beijing 100081, China

**Keywords:** yttria-stabilized zirconia (YSZ) powders, induction plasma spheroidization (IPS), thermal barrier coatings (TBCs), powder performance improvement

## Abstract

In this study, the induction plasma spheroidization (IPS) technique was adopted to improve the microstructure and properties of the traditional agglomerated ZrO_2_-7wt%Y_2_O_3_ (YSZ) powders used in thermal barrier coating (TBC) applications. Compared with agglomerated YSZ powders, IPS-treated powder has a more desirable microstructure, and the overall performance of the spray powders for TBC preparation is significantly improved. Specifically, IPS-treated powder has a dense, solid, defect-free, and chemically uniform microstructure, and its apparent density, flowability, and powder strength are significantly improved, which is believed to substantially enhance the coating performance when prepared with this IPS-treated powder.

## 1. Introduction

A gas turbine is a kind of power machinery that drives the impeller to rotate at high speed using the energy of a flowing working medium (high-temperature gas) and is widely used in civil aviation, military aircraft, ships, and vehicles [1,2]. Further improving the thrust-to-weight ratio and thermal efficiency of gas turbines by increasing the working temperature of the combustion chamber has become the goal of researchers. However, it poses a significant challenge to the high-temperature capability of the gas turbine components [3]. In the past few decades, due to the successful application of thermal barrier coating (TBC) acting as a thermal insulation layer, the performance of gas turbines has been greatly improved [4].

Classic TBC comprises a metal bonding coat and a ceramic top layer [5,6,7,8]. Furthermore, yttrium-stabilized zirconia (YSZ) has become the most successful material in ceramic layers for its comprehensive properties [9,10,11,12]. However, the established consensus suggests that the TBC prepared using YSZ powders with a traditional preparation method (spray granulation combined with high-temperature sintering) often has problematic issues, such as insufficient high-temperature capability, low bonding strength, unfavorable phase transformation, and poor corrosion and erosion resistance, thus resulting in its premature failure; therefore, the operating temperature is strictly limited to below 1200 °C [13,14,15,16,17,18,19]. In this context, optimizing the YSZ powders for operating conditions with higher temperatures is urgently needed.

Among the technologies for treating powders, induction plasma spheroidization (IPS) is a powerful technique to enhance the overall properties of the powder [20,21,22,23]. The IPS process uses high temperature (~10,000 °C) and high energy density of the generated plasma to rapidly heat and melt or gasify the feedstock powders to eliminate micro-defects and impurities. Recently, researchers have established advanced models to study the inductively coupled plasma torch’s electromagnetic field, temperature field, and fluid field, which helps us better understand the spheroidization process [24,25]. The molten particles form spherical droplets under the effect of surface tension in the heating process and rapidly solidify to form spherical powders. Thus, the powders′ density and flowability could also be enhanced during the cooling process [26,27,28]. Ceramic powders have always been treated with this method to obtain powders with fewer micro-defects, high purity, high apparent density, enhanced powder strength, and excellent flowability [29,30,31]. However, to the best of our knowledge, no comprehensive study currently reveals the effect of IPS on the microstructure and properties of YSZ powders.

In this study, the IPS technique was adopted to treat the agglomerated YSZ powders using the traditional spray drying method, and the influence of IPS parameters on the microstructure and properties of agglomerated powders was investigated. Using parameter optimization, desirable IPS-treated powder with improved microstructure was obtained, and its overall property was found to be significantly enhanced, which is very promising in improving the performance of the corresponding TBC prepared with this powder.

## 2. Materials and Methods

Firstly, 7 wt% Y_2_O_3_-stabilized ZrO_2_ (7YSZ) agglomerated powders were prepared using the spray drying method. YSZ particles (99.9%, 1–3 µm, Yttrium oxide 7~7.5 wt.%, BGRIMM Advanced Materials Science and Technology Co., Ltd., Beijing, China) were used as raw material. Raw materials were calculated and weighed. Then, the oxides were mixed and ground via ball milling (GMJ/B, Xianyang Jinhong General Machinery Ltd., Xianyang, China), during which zirconia balls and deionized water were used as the milling medium. The mass ratio of powders: water: zirconia ball was controlled at 1:0.6:3, and a small amount of binder PVA was added. After 24 h of ball milling, a slurry with appropriate viscosity was removed and dry-sprayed with a spray-drying tower (LGZ-8, Wuxi Dongsheng Spray Drying Equipment Ltd., Wuxi, China). Detailed granulation parameters are listed in Table 1. Afterward, the agglomerated powder was calcined at 1000 °C for 3 h to remove the binder. For agglomerated powders used for IPS treatment, powders were first sieved to control the particle size ranging from 25 µm to 75 µm.

Then, the agglomerated YSZ powders were treated using the induction plasma system technique (Tek-80kW, TEKNA Plasma Systems Inc., Sherbrooke, QC, Canada). In the IPS process, many key parameters could affect the state of the powder when flowing in the plasma flame, among which the power and feed rate matter most. Here, the power remained unchanged, and two sets of experiments with variable feed rates were designed, and specific parameters are shown in Table 2.

For characterization, the surface and cross-sectional micrographs of the samples were examined using scanning electron microscopy (SEM, SU5000, Hitachi Ltd., Tokyo, Japan). Before SEM observation, samples were covered by a thin gold layer. Phase structure was identified using X-ray diffraction (XRD, Bruker D8 Advanced, Karlsruhe, Germany) with CuKα radiation. Data were digitally collected in 2θ range of 20°–80° under a continuous scan mode, with a scanning rate of 0.2°/s. The apparent density and flowability of the powders were tested using a Hall flowmeter funnel. The precise chemical compositions of the agglomerated and IPS-treated powders were determined using the inductively coupled plasma optical emission spectrometer (ICP-OES) method (725-ES, Agilent Technologies Inc., Santa Clara, CA, USA).

**Figure 1:**
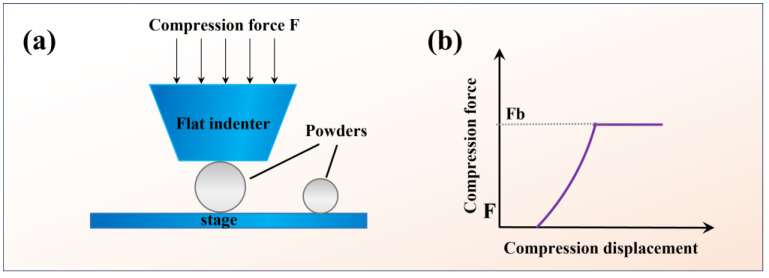
(**a**) Schematic drawing of the indentation process and (**b**) compression force and compression displacement curve.

A micro compression testing machine (SHIMADZU, Kyoto, Japan) was utilized to determine the particle strength [32]. The schematic drawing of the indentation process can be seen in Figure 1a. The compression force (F) rapidly grows as the indenter continues to crush the particle, leading to the particle’s breaking, as seen by the force curve (Figure 1b). The following formula can be used to calculate the compressive strength (*Cs*) from the force at the breaking point (*Fb*) [33]:

(1) $$Cs=C\;*\;Fb/(\pi \;*\;d^{2})$$

where *Cs* is the compressive strength (MPa), *Fb* is the compressive force at the breaking point (N), *d* is the particle’s diameter (μm), and *C* is a constant that, according to standard JIS R 1639-5, equals 2.48 [34]. For repeatability, at least five single particles with the size ranging from 30 to 50 µm were tested.

## 3. Results and Discussion

### 3.1. Characterization of the Agglomerated YSZ Powders

**Figure 2:**
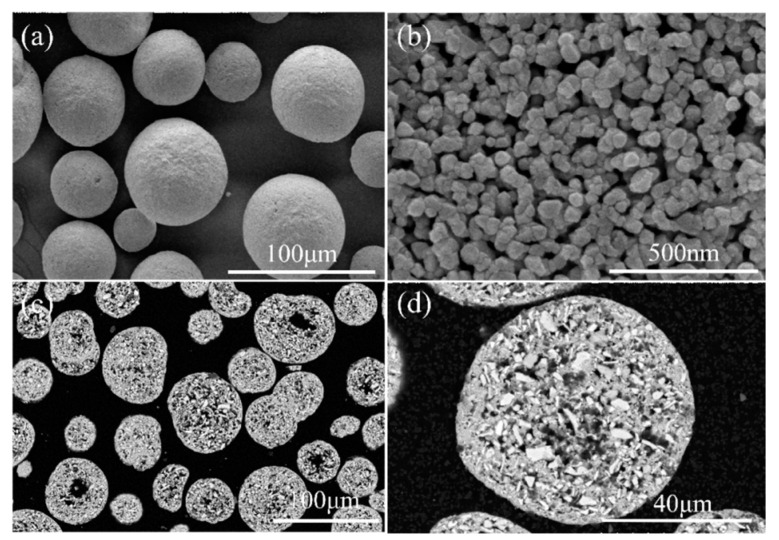
Microstructures of the agglomerated YSZ powders: (**a**) surface morphology with low magnification, (**b**) surface morphology of a single particle with high magnification, (**c**) cross-sectional morphology with low magnification, and (**d**) cross-sectional morphology of a single particle with high magnification.

**Figure 3:**
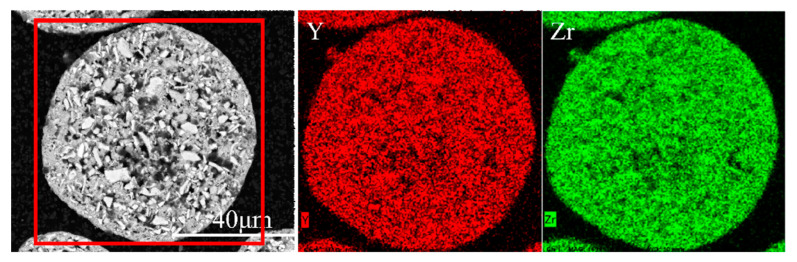
Elemental distribution of the agglomerated YSZ powder detected via EDS mapping.

**Figure 4:**
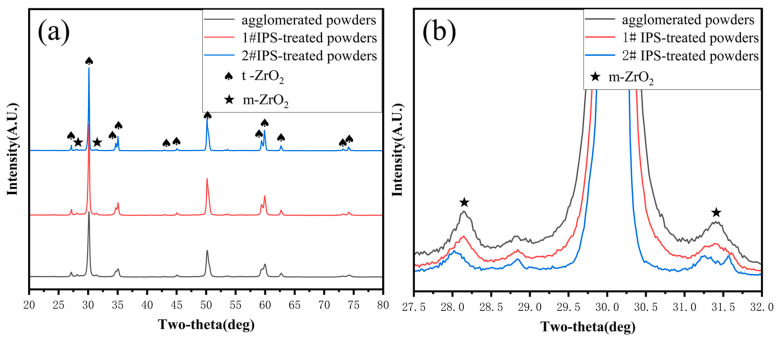
XRD diffractograms of the YSZ powders before and after IPS treatment: (**a**) 20–80° and (**b**) 27.5–32°.

The surface morphology of the agglomerated YSZ powder is shown in Figure 2a,b. At low magnification, the agglomerated powder exhibits a classic spherical shape with a particle size of 25–75 μm (Figure 2a). Further observation on the surface of a single particle reveals its loose structure, which is composed of numerous fine particles with a particle size of less than 100 nm (Figure 2b). The cross-sectional perspective (Figure 2c,d) shows that most powders have a solid structure composed of several nanoparticles, consistent with the observation made on the powder surface. A single particle was randomly picked, and elemental distribution was made. Figure 3 clearly shows that Y and Zr are relatively uniformly distributed within a single particle, with some pores not reflecting the elemental signals. The phase composition of the powder was also tested, and it was found that the agglomerated powder is mainly composed of desirable t-phase, with only a trace of m-phase remaining (Figure 4).

### 3.2. Characterization of the IPS-Treated YSZ Powders

Figure 4 shows the XRD patterns of YSZ powders before and after IPS treatment. After IPS treatment, it is found that the main component of IPS powder is t-phase while a small amount of m-phase still remains, which is the same as the agglomerated YSZ powders, indicating that IPS treatment does not change the phase structure of the powder (Figure 4a). Also, the intensities of the characteristic peaks ascribed to m phase ~28° were compared after normalizing the three XRD patterns (Figure 4b). It was found that the peak intensity of the m phase of the agglomerated powder was the highest, followed by the IPS-1 powder, and the IPS-2 powder had the weakest m phase peak intensity. This indicates that the IPS process could help to eliminate impurities and purify the phase structure. On the other hand, the chemical compositions of the agglomerated powder and IPS-treated powder were tested using ICP. It was found that the IPS-treated powder is purer and has a lower impurity content (Table 3) because the high temperature of the plasma torch burned out some impurities.

When the powder is fed into the plasma flame with a temperature of up to 10,000 K, it quickly melts from the surface to the inside under the high-temperature effect, allowing the powder to eliminate impurities, nanoparticles, and microscopic defects. This helps to improve the chemical purity, density, and, thus, the strength of the powder. Afterward, the powder leaves the flame flow. Under the effect of surface tension, it solidifies into the sphere when the temperature rapidly drops, which greatly helps improve the powder’s sphericity and fluidity. The impacts of IPS treatment on powder morphology and properties will be demonstrated in detail here.

**Figure 5:**
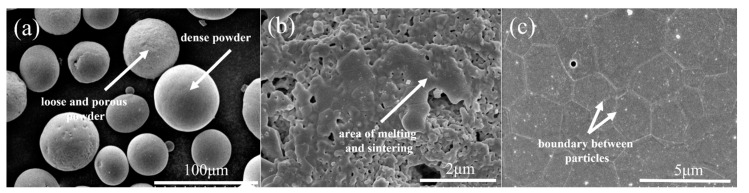
Surface morphology of the 1# IPS-treated YSZ powders: (**a**) overall morphology of the powders, (**b**) surface of the porous powders, and (**c**) surface of the dense powders.

**Figure 6:**
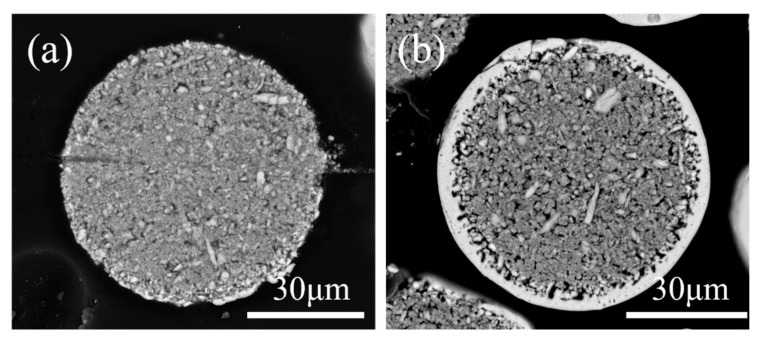
Cross-sectional morphology of the 1# IPS-treated YSZ powders: (**a**) cross-section of the porous powder and (**b**) cross-section of the dense powder.

Figure 5 firstly exhibits the microstructure of the 1# IPS-treated powders. Two distinct powders with completely different morphologies can be found (Figure 5a): one is loose and porous, seemingly similar to the original agglomerated powder, while the other has a dense structure. Observation on the surface of the porous powder (Figure 5b) suggests that the powders have undergone a sintering effect compared with the agglomerated powder to some extent but have hardly melted, indicating that the treatment effect of IPS is negligible in this case. Further examination of the cross-section (Figure 6a) suggests that the internal microstructure of the porous powder with a negligible melting degree does not change significantly compared with that of agglomerated powder. For the dense powder (Figure 5c), a dense and smooth surface could be clearly seen. The nanoparticles disappear, and large grains with clear grain boundaries form, indicating that the powder has seemingly undergone complete melting and re-solidification. However, its cross section suggests that only a dense shell layer with a thickness of less than 5 μm forms with the core remaining unchanged, indicating the insufficient melting degree. The above discussion reveals that under 1# IPS treatment, the powders could not fully melt and re-solidify, and the characteristics of the agglomerated powder have mainly been preserved.

Feeding rate and power are critical parameters in the IPS treatment process that influence the powder’s sintering effect and melting degree. The amount of powder delivered into the plasma jet per unit of time varies with the powder feeding rate modulation. The heat received by a single particle varies, and its melting state varies when a specific amount of heat absorption is assumed per unit of time. The lower the powder feeding rate is, the more complete the melting degree of the particles is and the denser the structure is. On the other hand, the plasma power determines the temperature of the plasma jet. The higher the power, the higher the heating level of the powders. Therefore, optimizing the powder feeding rate and plasma power is a reasonable way to obtain powders with a more dense structure. This kind of structure is conducive to improving the powder properties, and the performance of the TBC prepared using this powder.

**Figure 7:**
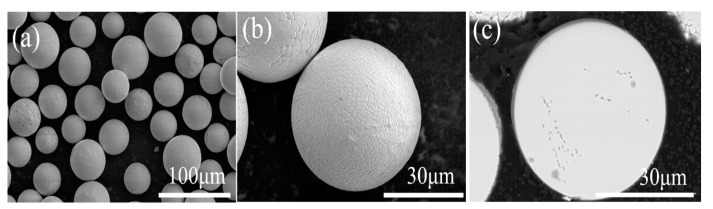
Morphology of the 2# IPS-treated YSZ powders: (**a**) overall morphology of the powders, (**b**) surface morphology, and (**c**) cross-sectional morphology.

**Figure 8:**
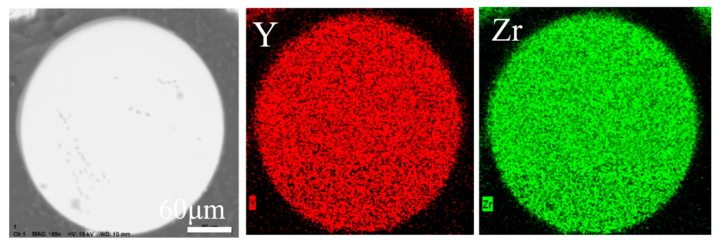
Elemental distribution of the cross-section of the 2# IPS-treated YSZ powders detected via EDS mapping.

Figure 7a shows the overall morphology of the YSZ powder treated with the 2# IPS process. Almost all powders have a dense surface shell, and the surface morphology of a single particle is shown in Figure 7b. The surface is dense and smooth. Further observation of the cross-section of the powder shows that the powder has a dense and solid microstructure with no nanoparticles remaining, indicating that by adjusting the powder feeding rate, the powders are fully melted and re-solidified, effectively eliminating the structural defects. The elemental distribution results via EDS are shown in Figure 8. Compared with the agglomerated powder, the uniformity of the distribution of Zr and Y atoms on the cross-section of YSZ powder is greatly improved because the plasma-treated powders have eliminated the microscopic defects such as pores inside.

The properties of the agglomerated powders and IPS-treated powders were tested and compared. The apparent density, flowability, and particle size distribution of the agglomerated powders and the IPS-treated YSZ powders are shown in Table 4. It can be found that after IPS treatment, the apparent density is significantly increased compared with the agglomerated powders, from 1.26 g/cm^3^ to 1.92 g/cm^3^ (1# IPS-treated) and 3.18 g/cm^3^ (2# IPS-treated). Also, the flow time is obviously decreased, from 62.74 s to 39.68 s (1# IPS-treated) and 21.46 s (2# IPS-treated), implying better flowability after IPS treatment. From the results of particle size distribution, the particle size of YSZ-agglomerated powder becomes significantly smaller after the IPS process, and the shrinkage trend of densification is obvious. These results show that the IPS treatment is an effective method to increase the density and flowability of the YSZ powders, decrease the particle size, and narrow the powder size distribution. It should be noted that the enhancement of the powder performance via IPS treatment technology depends on the melting state and corresponding microstructure of the powder. Only powders that undergo a complete melting and re-solidification process can achieve the most significant improvement in properties.

**Figure 9:**
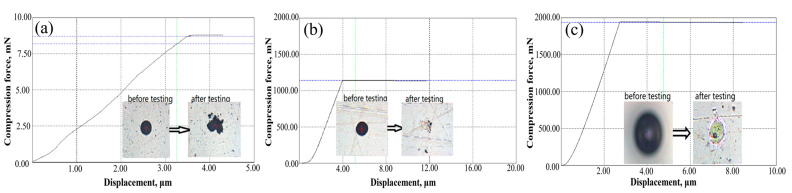
Typical compression force curves and the morphology of YSZ particle: (**a**) agglomerated powders; (**b**) 1# IPS-treated powders; and (**c**) 2# IPS-treated powders.

Figure 9 shows the typical compression force curves of YSZ powders and the typical powder morphology before and after the test. Moreover, the compressive strength values of agglomerated YSZ powders and IPS-treated YSZ powders are shown in Table 5. An indentation test was first conducted on YSZ-agglomerated powders, and it was found that the powder quickly collapsed. After compression, the powder flattened, and the average compression strength was only 4.8 MPa. This is because under the calcination process with relatively low temperatures, the nanoparticles that make up the powder do not undergo significant growth, and the interconnection between the nanoparticles is also weak.

However, when we examined the compression force cures and morphology of the YSZ powder after the indentation test of the powder treated using the IPS process (Figure 9b,c), it was found that as the displacement of the indenter increases, a stress plateau quickly appears, indicating that the powder has a strong resistance to resist collapse. Moreover, IPS-2# powder exhibits the stress plateau faster, and a higher plateau is obtained, indicating higher powder strength and toughness (Figure 9c). After indentation, some powders were splashed, resulting in an improvement in the powder’s internal strength. When the indentation could not easily crush the powder, the powder would slide relative to the plane under pressure and be splashed away. In this case, it is difficult to determine the compressive strength of the powder. Thus, for IPS-treated powders, compressive strength was only collected from those powders that collapsed instead of splashing away. It should be noted that the obtained compression strength must be significantly lower than its actual value. Nevertheless, the compressive strength of the powder treated using IPS is still much higher than that of the agglomerated powder, which can be attributed to the fact that IPS treatment effectively eliminates all nanoparticles and microscopic defects.

When spray powders are used to prepare coatings, the coating is bound to inherit certain characteristics of the spray powder to some extent. Specifically, when the agglomerated powders with loose structures composed of numerous nanoparticles serve as the spray powder, the obtained coating is inevitably very likely to have un-melted nanoparticles and porous structures with high porosity, which is very detrimental to the bonding strength and thermal cycling lives. Therefore, it is foreseen that by using the IPS-treated powders with dense and defect-free microstructure, the bonding strength and the cycling performance of the resulting coating are highly likely to be greatly improved, and this is our future work.

## 4. Conclusions

This study adopted the IPS technique to treat the traditional agglomerated YSZ powders to improve their comprehensive performance in TBC applications. It was found that the IPS parameters had a dominant effect on the microstructure and properties of the powder by regulating the melting state of the powder in the plasma flame. The IPS-treated powder with full melting and re-solidification was obtained through parameter optimization, the microstructure of which was transformed from the loose state of the agglomerated powders to the dense and defect-free state. Correspondingly, the comprehensive performance of the powder was greatly improved compared with the agglomerated powders. Specifically, the apparent density was increased from 1.26 g/cm^3^ to 3.18 g/cm^3^, and the average compressive strength was increased from 4.8 MPa to 625.6 MPa. Also, the chemical uniformity and flowability of the powder was significantly improved. The improvement of powder properties must be reflected in the coating performance, and this effect will be discussed in our future study.

## Figures and Tables

**Table 1:** Parameters of spray drying for agglomerated YSZ powders.

Parameters	Values
Inlet temperature	300 °C
Outlet temperature	120 °C
Atomizer rotary rate	40 Hz
Feed Pump rate	30 rpm

**Table 2:** Parameters for IPS treatment.

Series No.	Carrier Gas (Ar) (slpm)	H_2_ Flow (slpm)	Ar Flow (slpm)	Reactor Pressure (psia)	Powder Feed Rate (g/min)	Power (kW)
1#	95	15	30	15	100	80
2#	95	15	30	15	40	80

**Table 3:** Chemical composition of YSZ powders using ICP.

Content	Y_2_O_3_ (wt%)	Al_2_O_3_ (wt%)	SiO_2_ (wt%)	TiO_2_ (wt%)	Cl^−^ (wt%)
Agglomerated YSZ powder	7.32	0.042	0.038	0.012	0.036
IPS-treated YSZ powder (2#)	7.28	0.019	0.018	0.006	0.010

**Table 4:** Apparent density and flowability of the agglomerated and IPS-treated YSZ powders.

	Property	Apparent Density (g/cm^3^)	Flowability (s/50 g)	Particle Size Distribution (μm)
Powder				
Agglomerated YSZ powder		1.26	62.74	D10 = 36.0 D50 = 56.0 D90 = 84.4
1# IPS-treated YSZ powder		1.92	39.68	D10 = 35.9 D50 = 51.6 D90 = 72.4
2# IPS-treated YSZ powder		3.18	21.46	D10 = 35.3 D50 = 43.9 D90 = 55.2

**Table 5:** Compressive strength of the agglomerated and IPS-treated YSZ powders.

Type of Powder	Strength Range (MPa)	Average Compressive Strength (MPa)
Agglomerated YSZ powders	2~8	4.8
1# IPS-treated YSZ powders	80~190	126.3
2# IPS-treated YSZ powders	420~750	625.6

## Data Availability

Data are contained within the article.
